# Genome-Wide Identification and Analysis of Grape Aldehyde Dehydrogenase (ALDH) Gene Superfamily

**DOI:** 10.1371/journal.pone.0032153

**Published:** 2012-02-15

**Authors:** Yucheng Zhang, Linyong Mao, Hua Wang, Chad Brocker, Xiangjing Yin, Vasilis Vasiliou, Zhangjun Fei, Xiping Wang

**Affiliations:** 1 College of Horticulture, Northwest A&F University, Key Laboratory of Horticultural Plant Biology and Germplasm Innovation in Northwest China, Ministry of Agriculture, Yangling, Shaanxi, People's Republic of China; 2 College of Enology, Northwest A&F University, Shaanxi Engineering Research Center for Viti-Viniculture, Yangling, Shaanxi, People's Republic of China; 3 Boyce Thompson Institute for Plant Research, Cornell University, Ithaca, New York, United States of America; 4 Molecular Toxicology and Environmental Health Sciences Program, Department of Pharmaceutical Sciences, University of Colorado Anschutz Medical Center, Aurora, Colorado, United States of America; 5 USDA Robert W. Holley Center for Agriculture and Health, Ithaca, New York, United States of America; 6 State Key Laboratory of Crop Stress Biology in Arid Areas, Northwest A&F University, Yangling, Shaanxi, People's Republic of China; Instituto de Biología Molecular y Celular de Plantas, Spain

## Abstract

**Background:**

The completion of the grape genome sequencing project has paved the way for novel gene discovery and functional analysis. Aldehyde dehydrogenases (*ALDHs*) comprise a gene superfamily encoding NAD(P)^+^-dependent enzymes that catalyze the irreversible oxidation of a wide range of endogenous and exogenous aromatic and aliphatic aldehydes. Although *ALDHs* have been systematically investigated in several plant species including *Arabidopsis* and rice, our knowledge concerning the *ALDH* genes, their evolutionary relationship and expression patterns in grape has been limited.

**Methodology/Principal Findings:**

A total of 23 *ALDH* genes were identified in the grape genome and grouped into ten families according to the unified nomenclature system developed by the ALDH Gene Nomenclature Committee (AGNC). Members within the same grape *ALDH* families possess nearly identical exon-intron structures. Evolutionary analysis indicates that both segmental and tandem duplication events have contributed significantly to the expansion of grape *ALDH* genes. Phylogenetic analysis of ALDH protein sequences from seven plant species indicates that grape ALDHs are more closely related to those of *Arabidopsis*. In addition, synteny analysis between grape and *Arabidopsis* shows that homologs of a number of grape *ALDHs* are found in the corresponding syntenic blocks of *Arabidopsis*, suggesting that these genes arose before the speciation of the grape and *Arabidopsis*. Microarray gene expression analysis revealed large number of grape *ALDH* genes responsive to drought or salt stress. Furthermore, we found a number of *ALDH* genes showed significantly changed expressions in responses to infection with different pathogens and during grape berry development, suggesting novel roles of *ALDH* genes in plant-pathogen interactions and berry development.

**Conclusion:**

The genome-wide identification, evolutionary and expression analysis of grape *ALDH* genes should facilitate research in this gene family and provide new insights regarding their evolution history and functional roles in plant stress tolerance.

## Introduction

Plants are exposed to many types of abiotic stresses during their life-cycle, such as drought, salinity, and low temperature [1]. Plants adapt to abiotic stresses by the expression of a wide range of stress-responsive genes, which are thought to play key roles in stress tolerance and survival [2]. Endogenous aldehyde molecules are intermediates or by-products of several fundamental metabolic pathways, and they are also excessively generated in response to environmental stresses such as salinity, dehydration, desiccation, cold and heat shock [3], [4]. Although aldehydes are associated with common biochemical pathways, the compounds can be extremely toxic when produced in excess because of their inherent chemical reactivity [5]. Aldehyde dehydrogenases (*ALDHs*) comprise a gene superfamily encoding NAD(P)^+^-dependent enzymes that catalyze the irreversible oxidation of a wide range of endogenous and exogenous aromatic and aliphatic aldehydes [6]. *ALDHs* are responsible for efficient detoxification of aldehydes by converting them to carboxylic acids [6]. Additionally, they also carry out a broad range of other metabolic functions including (i) participating in intermediary metabolism, such as amino acid and retinoic acid metabolism; (ii) providing protection from osmotic stress by generating osmoprotectants, such as glycine betaine [7], [8]; and (iii) generating NAD(P)H [9]. In plants, the *ALDH* genes are expressed throughout various different tissues and in response to a wide variety of stressors [10], [11].

Throughout all taxa, *ALDHs* have been classified into 24 distinct families to date. These families are numbered according to the criteria established by the ALDH Gene Nomenclature Committee (AGNC) [12]. ALDHs with amino acid sequences that are more than 40% identical to previously identified ALDH sequences comprise a family, while those with sequences more than 60% identity comprise a subfamily. ALDHs with sequences less than 40% identity represent a new family. Among the 24 ALDH families, 14 (ALDH2, ALDH3, ALDH5, ALDH6, ALDH7, ALDH10, ALDH11, ALDH12, ALDH18, ALDH19, ALDH21, ALDH22, ALDH23 and ALDH24) contain members from plant species and seven (ALDH11, ALDH12, ALDH19, ALDH21, ALDH22, ALDH23 and ALDH24) are unique to plants. Much work has been carried out on the *ALDH* gene family in prokaryotes and mammals [6], [13], whereas the research on plant *ALDHs* is relatively limited. Furthermore, most of the analyses on plant *ALDHs* have been performed in model species such as *Arabidopsis* [6] and rice [11], with little attention paid to woody species like grape.

Grapevine (*Vitis vinifera*) is economically the most important perennial fruit crop worldwide, and the fourth angiosperm species, the second woody species, and the first fruit crop to have a fully sequenced genome [14], [15]. Compared to other perennials, the genome size of *V. vinifera* is relatively small, 475 Mb, which is similar to rice (*Oryza sativa*, 430 Mb) [16], barrel medic (*Medicago truncatula*, 500 Mb, http://medicago.org/) and black cottonwood poplar (*Populus trichocarpa*, 465 Mb) [17]. In addition, the grapevine genome has not undergone a recent whole genome duplication (WGD), thus enabling the discovery of ancestral traits and genetic divergence occurring during the course of flowering plant evolution [14]. The release of grape genome data allows us for the first time to carry out the genome-wide identification and analysis of *ALDH* gene families in a woody species. Here we systematically identified 23 *ALDH* genes belonging to ten different families in the grape genome. Phylogenetic and synteny analyses revealed segmental and tandem duplication events have contributed to grape *ALDH* evolution. We further analyzed expression profiles of grape *ALDH* genes under various abiotic and biotic stresses, in response to different phytohormone treatments, and during berry development and ripening, through mining publicly available microarray datasets. The results obtained from our study provided a foundation for evolutionary and functional characterization of *ALDH* gene families in grape and other plant species.

## Results and Discussion

### Genome-wide identification of *ALDH* gene families in grape

From the grape genome, we identified a total of 23 putative *ALDH* genes and grouped them into ten families based on their protein sequence identities (Table 1). Eight of the ten grape *ALDH* families are represented by more than one gene (*ALDH2*, three members; *ALDH*3, four members; *ALDH*5 and *ALDH*6, three members; and *ALDH*7, *ALDH*10, *ALDH*11, and *ALDH*18, two members), whereas the remaining two (*ALDH*12 and *ALDH*22) are single gene families. Among the 23 grape *ALDH* genes, one (*VvALDH18B1*) corresponds to a previously published gene (GenBank Acc#: AJ005686) [18], ten are supported by cDNA sequences that contain the full coding regions (their corresponding GenBank Acc# are *VvALDH2B8*: FQ382277; *VvALDH5F1*: FQ382545; *VvALDH10B1*: FQ384094; *VvALDH3H1*: FQ391752; *VvALDH2B4*: FQ391821; *VvALDH11B1*: FQ392316; *VvALDH2B9*: FQ392766; *VvALDH10A9*: FQ393912; *VvALDH3H5*: FQ394868; *VvALDH6B3*: FQ394961; *VvALDH7B5*: FQ395151), and another ten are supported by at least one EST sequence available in GenBank dbEST database; while only one (*VvALDH7D1*) is lack of support by EST or mRNA sequences.

**Table 1:** Grape *ALDH* genes and superfamilies.

Family	Gene ID	Gene Locus ID	Accession No.	Putative function	CDS (bp)	ORF (aa)
Family 2	VvALDH2B4_v1	GSVIVG01007784001	XM_002283096	Mitochondrial ALDH	1617	538
	VvALDH2B4_v2	GSVIVG01007784001	JN381165	Cytosolic ALDH	1434	477
	VvALDH2B4_v3	GSVIVG01007784001	JN381166	Mitochondrial ALDH	1578	525
	VvALDH2B8	GSVIVG01020224001	XM_002263443	Mitochondrial ALDH	1617	538
	VvALDH2B9	GSVIVG01032500001	XM_002274827	Mitochondrial ALDH	1608	535
Family 3	VvALDH3F1	GSVIVG01018842001	XM_002273322	Variable substrate ALDH	1458	485
	VvALDH3H1	GSVIVG01008845001	XM_002285830	Variable substrate ALDH	1467	488
	VvALDH3H5	GSVIVG01022356001	XM_002273694	Variable substrate ALDH	1467	488
	VvALDH3J1	GSVIVG01025276001	XM_002285430	Variable substrate ALDH	1458	485
Family 5	VvALDH5F1	GSVIVG01036719001	XM_002265478	Succinic semialdehyde dehydrogenase	1593	530
	VvALDH5F2	GSVIVG01036720001	XM_002265366	Succinic semialdehyde dehydrogenase	1482	493
	VvALDH5F3	GSVIVG01036721001	XM_002265318	Succinic semialdehyde dehydrogenase	1476	491
Family 6	VvALDH6B3	GSVIVG01000336001	XM_002266354	Methylmalonate semi-aldehyde dehydrogenase	1620	539
	VvALDH6B5	GSVIVG01000338001	XM_002266580	Methylmalonate semi-aldehyde dehydrogenase	1716	571
	VvALDH6B7	GSVIVG01003951001	XM_002266343	Methylmalonate semi-aldehyde dehydrogenase	3096	1031
Family 7	VvALDH7B5	GSVIVG01015062001	XM_002278057	Antiquitin	1527	508
	VvALDH7D1	GSVIVG01016734001	XM_002272508	Antiquitin	1593	530
Family 10	VvALDH10A9	GSVIVG01007829001	XM_002283654	Betaine-aldehyde dehydrogenase	1512	503
	VvALDH10B1	GSVIVG01032588001	XM_002281948	Betaine-aldehyde dehydrogenase	1500	499
Family 11	VvALDH11A3	GSVIVG01035891001	XM_002285250	NADH-dependent glyceraldehyde-3-Phosphate dehydrogenase	1491	496
	VvALDH11B1	GSVIVG01023590001	XM_002279338		1491	496
Family 12	VvALDH12A1	GSVIVG01008047001	XM_002273533	Δ^1^-pyrroline-5-carboxylate dehydrogenase	1668	555
Family 18	VvALDH18B1	GSVIVG01016467001	XM_002282319	Δ^1^-pyrroline-5-carboxylate synthetase	2289	762
	VvALDH18B3	GSVIVG01034097001	XM_002273220	Δ^1^-pyrroline-5-carboxylate synthetase	2145	714
Family 22	VvALDH22A1	GSVIVG01035003001	XM_002277707	Novel ALDH	1782	593

We have previously identified three alternatively spliced variants of *VvALDH2B4* in wild Chinese grape, *V. pseudoreticulata* [19]. We subsequently confirmed these three splice variants using RT-PCR in other grape cultivars including Pinot Noir, Chardonnay, *V. quinquangularis* clone ‘shang-24’, *V. pseudoreticulata* clone ‘Hunan-1’, and *V. piazezkii* Maxim. clone ‘Meixian-6’. We named the three alternatively spliced transcripts of *VvALDH2B4* as *VvALDH2B4*_v1, *VvALDH2B4*_v2 and *VvALDH2B4*_v3 according to nomenclature guidelines for alternative transcriptional variants of *ALDH* genes [20]. *VvALDH2B4*_v1 and *VvALDH2B4*_v3 have different 3′ splice acceptor sites in the third exon, while *VvALDH2B*_v2 has an intron retention which leads to a different translation initiation site (Fig. S1). As a result, *VvALDH2B4*_v2 encodes a 477-residue protein with NH_2_-terminal truncated, compared to 538- and 525-residue of *VvALDH2B4*_v1 and *VvALDH2B4*_v3, respectively. To date all characterized plant species possess two types of ALDH2 proteins: mitochondrial and cytosolic [6], [21], [22] and they all contain two mitochondrial ALDH2 proteins [6], [23]. Analysis with the PSORT program [24] showed that *VvALDH2B4*_v1 and *VvALDH2B4*_v3 protein sequences each contained a predicated N-terminal mitochondrial targeting signal, whereas *VvALDH2B4*_v2 was predicted to function in cytoplasm. In mammals, these enzymes play a role in detoxifying lipid peroxidation-derived aldehydes produced during oxidative stress as well as acetaldehyde produced during ethanol metabolism [25]. However, the specific functions of both mitochondrial and cytosolic ALDH2 proteins in plants remain to be determined.

### Comparative analysis of *ALDH* gene families from various organisms

In the present study, we summarized numbers of gene family members for each individual *ALDH* family in *V. vinifera* and seven other plant species (*A. thaliana* [6], *Zea mays* [21], *O. sativa* [22], *Physcomitrella patens*, *Chlamydomonas reinhardtii* and *Ostreococcus tauri* [26]), three mammals (*Homo sapiens*, *Mus musculus* and *Rattus norvegicus*; http://www.aldh.org/), and fungi [27] (Table 2). Plant *ALDHs* are present in 13 families: *ALDH2*, *ALDH3*, *ALDH5*, *ALDH6*, *ALDH7*, *ALDH10*, *ALDH11*, *ALDH12*, *ALDH18*, *ALDH21*, *ALDH22*, *ALDH23* and *ALDH24*. *ALDH19* is also unique to plants and to date, has only been identified within the tomato genome and is thought to encode a γ-glutamyl phosphate reductase which may play a role in the biosynthesis of proline from glutamate [28]. *ALDH21* and *ALDH23* are unique to mosses and *ALDH24* is unique to *C. reinhardtii*. Grape and other studied vascular plants share ten common core *ALDH* families (*ALDH2*, *ALDH3*, *ALDH5*, *ALDH6*, *ALDH7*, *ALDH10*, *ALDH11*, *ALDH12*, *ALDH18*, and *ALDH22*), suggesting that these ten families evolved prior to the monocot/eudicot divergence. Eight of the ten core families (*ALDH2*, *ALDH3*, *ALDH5*, *ALDH6*, *ALDH10*, *ALDH11*, *ALDH12* and *ALDH22*) are also shared by terrestrial plants and algae, suggesting that these families have ancient origins predating the transition of aquatic plants onto land.

**Table 2:** Number of *ALDH* family members identified in various organisms.

Organism	ALDH family																							
	1	2	3	4	5	6	7	8	9	10	11	12	13	14	15	16	17	18	19	20	21	22	23	24
*V. vinifera*	−	3	4	−	3	3	2	−	−	2	2	1	−	−	−	−	−	2	−	−	−	1	−	−
*A. thaliana*	−	3	3	−	1	1	1	−	−	2	1	1	−	−	−	−	−	2	−	−	−	1	−	−
*Z.mays*	−	6	5	−	2	1	1	−	−	3	1	1	−	−	−	−	−	3	−	−	−	1	−	−
*O .sativa*	−	5	5	−	1	1	1	−	−	2	1	2	−	−	−	−	−	2	−	−	−	1	−	−
*P. patens*	−	2	5	−	2	1	1	−	−	1	5	1	−	−	−	−	−	−	−	−	1	−	1	−
*C. reinhardtii*	−	1	−	−	2	1	−	−	−	1	1	1	−	−	−	−	−	−	−	−	−	−	−	1
*O. tauri*	−	−	1	−	1	−	−	−	−	1	1	1	−	−	−	−	−	−	−	−	−	1	−	−
*H. sapiens*	6	1	4	1	1	1	1	1	1	−	−	−	−	−	−	1	−	1	−	−	−	−	−	−
*M. musculus*	7	1	4	1	1	1	1	1	1	−	−	−	−	−	−	1	−	1	−	−	−	−	−	−
*R. norvegicus*	7	1	4	−	1	1	1	1	1	−	−	−	−	−	−	1	−	1	−	−	−	−	−	−
*Fungi*	+	−	−	+	+	−	−	−	−	+	−	−	−	+	+	+	−	+	−	−	−	−	−	−

+ and − represent presence and absence, respectively, of the ALDH gene family in corresponding organisms.

It is worth noting that several previous studies indicated that *A. thaliana* genome lacks the *ALDH18* family [6], [21], [22], while other reports indicated the existence of two unique *ALDH18* genes in *Arabidopsis* genome [29], [30]. In this study, we performed a search for *ALDH18* genes in ‘The *Arabidopsis* Information Resource’ (TAIR, http://www.arabidopsis.org/) and confirmed that *A. thaliana* genome does contain two *ALDH18* genes located on chromosome 2 and 3, respectively.

It is worth noting that the apparent lack of *ALDH1* and *ALDH4* gene family members in plants is the result of nomenclature errors made when the genes were originally identified. The plant *ALDH2* genes should be included in the *ALDH1* family according to AGNC nomenclature guidelines. Both *ALDH4* and *ALDH12* encode delta-1-pyrroline-5-carboxylate dehydrogenases which play an important role in the degradation of proline to glutamate [31]. They also should be grouped into one single family.

*ALDHs* have been reported to play important roles in plant responses to various environmental stresses [6]. Plants, especially the higher plants like *V. vinifera* and *Z. mays*, seem to have more *ALDH* genes than animals and fungi. Unlike mammals, plants can not move and are therefore more susceptible to environmental insults, as a result they may require additional stress-response proteins such as ALDHs, to protect them when exposed to stress conditions. Compared to other well characterized plant *ALDHs*, grape *ALDH* families are the second most expanded with 23 genes, compared to 24 in *Z. mays*, 21 in *O. sativa*, 16 in *A. thaliana*, 20 in *P. patens*, eight in *C. reinhardtii*, and six in *O. tauri*.

**Figure 1:**
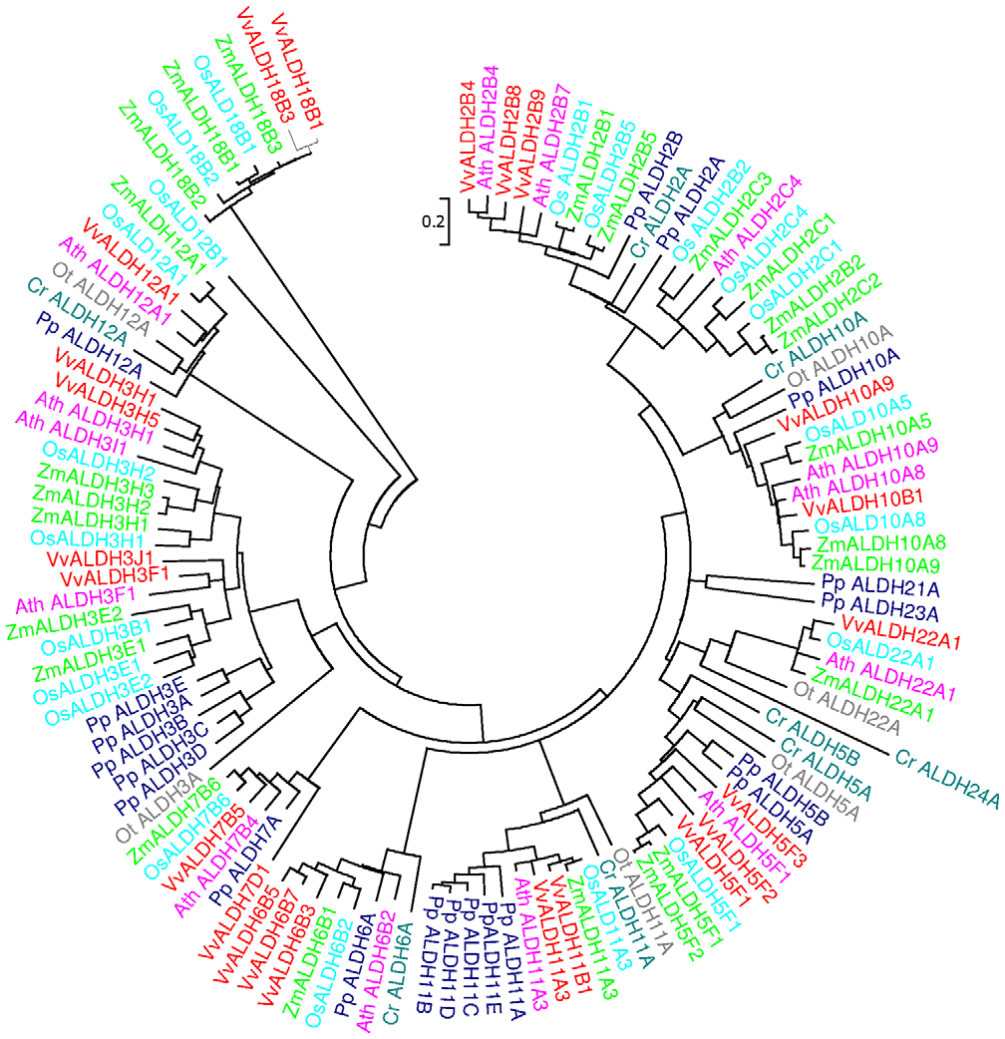
Phylogenetic analysis of grape and other plant ALDHs. Phylogenetic tree was constructed with ALDH protein sequences from *V. vinifera* (Vv), *Z. mays* (Zm), *O. sativa* (Os), *A. thaliana* (Ath), *P. patens* (Pp), *C. reinhardtii* (Cr), and *O. Tauri* (Ot).

We then extracted protein sequences of *ALDH* genes identified in *V. vinifera* and six other plant species, including *A. thaliana* [6], *P. patens*, *C. reinhardtii*, *O. tauri* [26], *Z. mays* [21] and *O. sativa* [22] and constructed a phylogenetic tree (Fig. 1). The tree indicated that the majority of *V. vinifera ALDH* families are more closely related to those in *A. thaliana*. *ALDH* genes from lower plants *P. patens*, *C. reinhardtii* and *O.tauri* diverged early on from their homologues in higher plants. This was followed by a relatively recent monocot/eudicot split. The result is consistent with the current understanding of plant evolutionary history.

As expected ALDH proteins from same families tend to cluster together. Though evolutionary relationships could not be clarified for all families, the phylogenetic analysis revealed some interesting observations. ALDH18, for example, is the phylogenetically most distantly related family. This is consistent with previous research in rice which indicated that two OsALDH18 proteins had the greatest degree of sequence divergence from other ALDH families and did not contain the conserved ALDH active sites [11].

### Phylogenetic and structural analysis of grape *ALDH* genes

**Figure 2:**
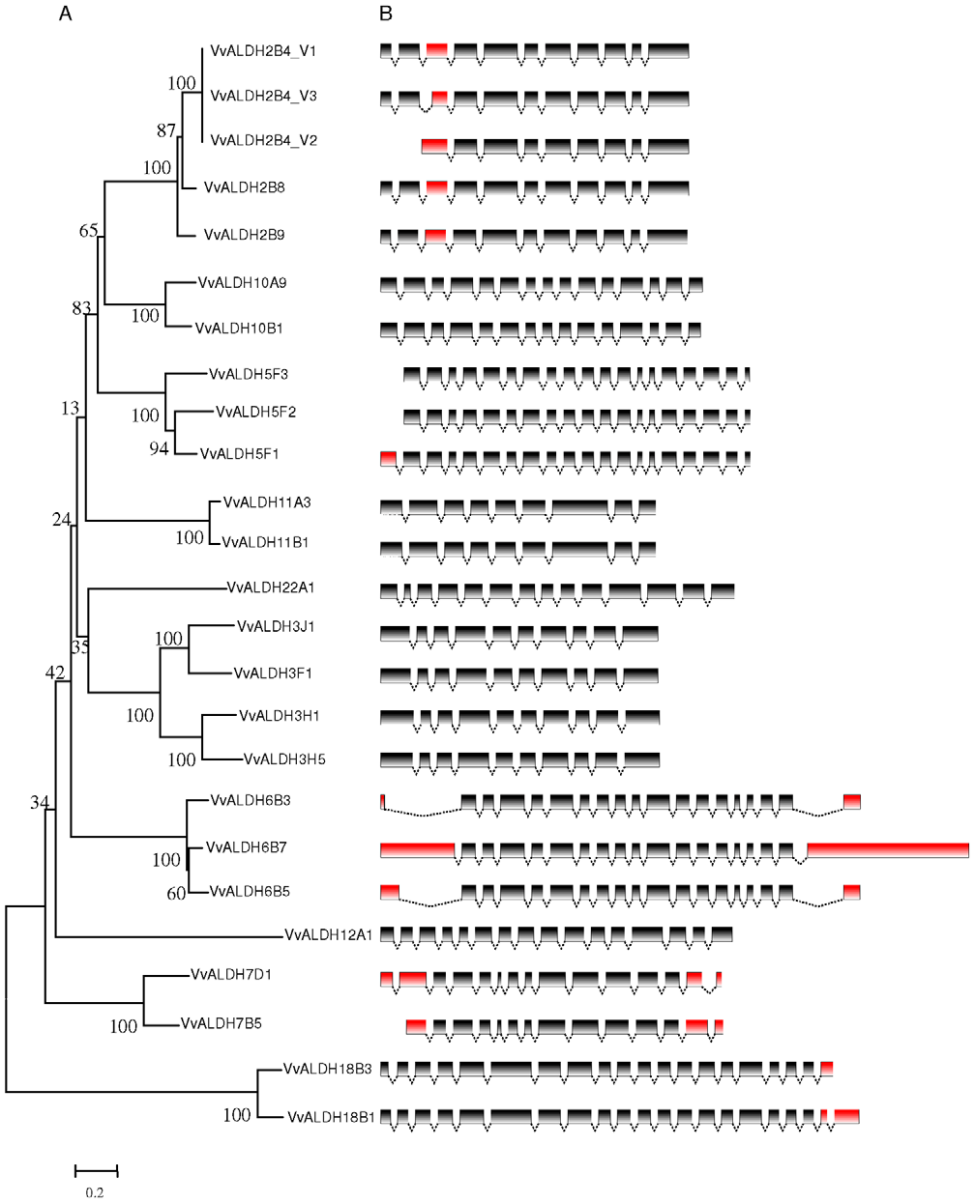
Phylogenetic analysis (A) and exon-intron structures (B) of grape *ALDH* genes. Numbers above or below branches of the tree indicate bootstrap values. Only coding exons, represented by black or red boxes, were drawn to scale. Dashed lines connecting two exons represent introns. Exons with different structures among the *ALDH* genes in same families were marked in red.

We constructed the phylogenetic tree of the 23 grape *ALDH* genes based on their amino acid sequences (Fig. 2a). The topology was similar to that constructed with *ALDH* genes from the seven plant species (Fig. 1) and, similarly, ALDH proteins from the same families are clustered together. Furthermore, the exon-intron structures of the *ALDH* genes were examined to gain more insights into their possible structural evolution. Exon-intron structural divergence within families plays a pivotal role in the evolution of multiple gene families. Our result showed that genes in the same family generally had similar exon-intron structures. *ALDH* genes in each of the families 2, 3, 6, 10 and 11 have the same number of exons and also exhibited nearly identical exon lengths, except for the first and last exons of the *ALDH6* genes (Fig. 2b). The high degree of sequence identity and similar exon-intron structures of *ALDH* genes within each family suggests that grape ALDH families have undergone gene duplications throughout evolution, resulting in *ALDH* gene families containing multiple copies that are partially or completely overlapping in function. A previous study demonstrated that *ALDH* genes from rice and *Arabidopsis* had highly conserved exon-intron structures [11]. In this study, we also compared the exon-intron structures of *ALDH* genes identified in the grape genome with those found in *Arabidopsis* and rice. The results indicated that the exon-intron structures were not only conserved within a species but also conserved across these three species (data not shown). Nonetheless, we did identify losses or gains of exons during the evolution of several *ALDH* genes. One such example is the *ALDH5* gene family. *ALDH5* genes in rice and *Arabidopsis,* as well as *VvALDH5F1* in grape, all have 20 exons; whereas grape *VvALDH5F2* and *VvALDH5F3* contain 19 exons, losing the first exon during evolution. Other examples include grape *VvALDH7D1* and *VvALDH18B1*, which have acquired one additional exon in 5′- and 3′-end, respectively, during evolution.

### Expansion patterns of *ALDH* gene families in grape

**Figure 3:**
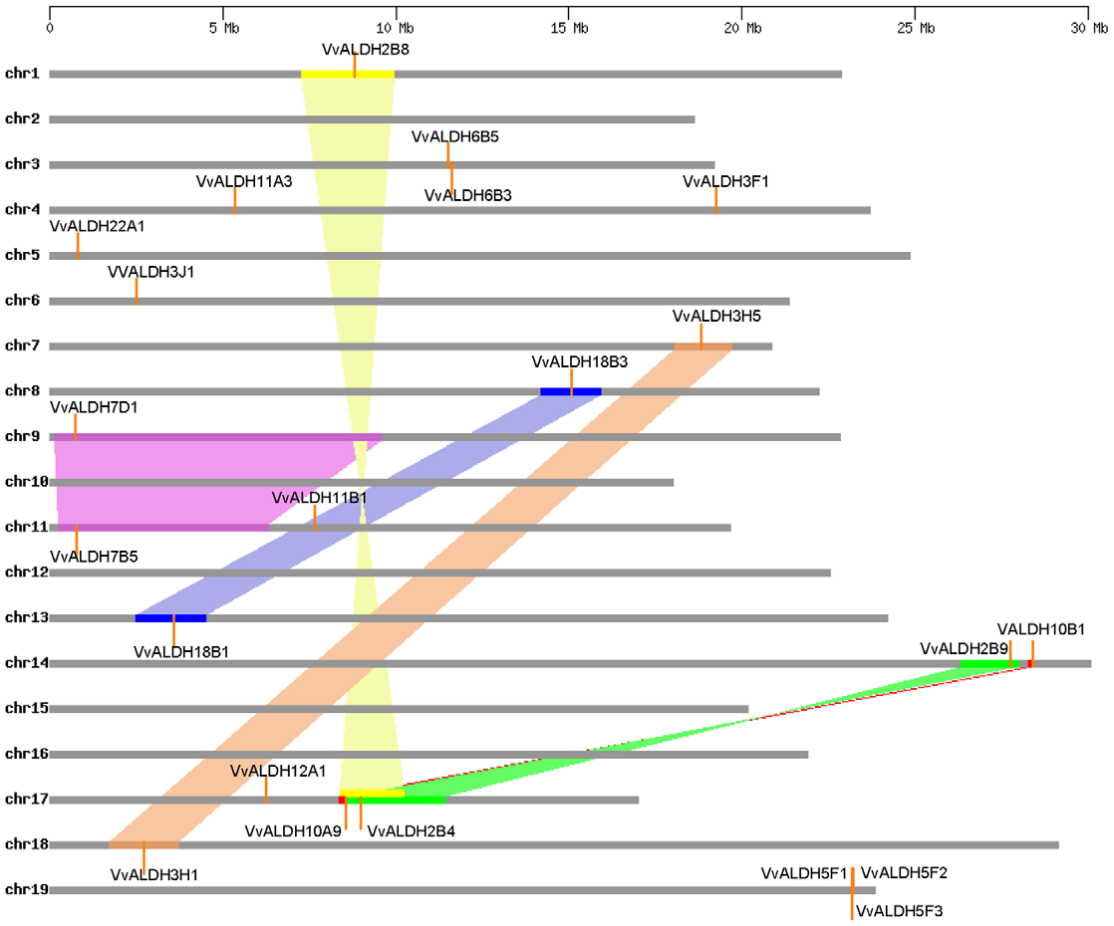
Distribution and synteny of *ALDH* genes on grape chromosomes. Chromosomes 1–19 (chr1–19) are depicted as horizontal gray bars. *ALDH* genes are indicated by vertical orange lines. Colored bars denote syntenic regions of the grape genome; the twisted colored bar indicates that the syntenic regions are in reverse orientation. *VvALDH6B7*, which is not assigned to any known chromosomes, is not shown.

Segmental and tandem duplications are two of the main reasons for gene family expansions [32]. Two tandem *ALDH* gene duplications have been reported in rice (*OsALDH2-1*/*OsALDH2-2* and *OsALDH3-1*/*OsALDH3-2*) [11]. In the present study, we also identified tandem duplications in two grape *ALDH* gene families (*VvALDH5F1*/*VvALDH5F2*/*VvALDH5F3* and *VvALDH6B3*/*VvALDH6B5*) (Fig. 3). We then examined the duplicated blocks within the grape genome and found that 11 grape ALDH genes from five families (*VvALDH2B4/VvALDH2B9*/*VvALDH2B8*, *VvALDH3H1*/*VvALDH3H5*, *VvALDH7B5*/*VvALDH7D1*, *VvALDH10A9*/*VvALDH10B1*, and *VvALDH18B1*/*VvALDH18B3*) were located in six pairs of duplicated genome regions (Fig. 3). In summary, seven out of eight grape multi-member *ALDH* gene families (Table 2) are associated with either segmental or tandem duplication events, indicating that segmental and tandem duplications have played important roles in the expansion of grape *ALDH* genes.

### Evolutionary relationship of *ALDH* gene families between grape and *Arabidopsis*

By comparing the sequences of all genes between genomes from different taxa and within each genome, it is, in principle, possible to reconstruct the evolutionary history of each gene in its entirety (within the set of sequenced genomes) [33]. To further explore the origin and evolutionary process of grape *ALDH* genes, we analyzed the comparative synteny map between grape and *Arabidopsis* genomes. Genomic comparison is a quick way to transfer genomic knowledge acquired in one taxon to a less-studied taxon [34]. *Arabidopsis* is the most important model plant species and the functions of most *Arabidopsis ALDH* genes have been well characterized. Thus, through comparative genomics analysis we could confidently infer the functions of grape *ALDHs* based on their *Arabidopsis* homologues.

**Figure 4:**
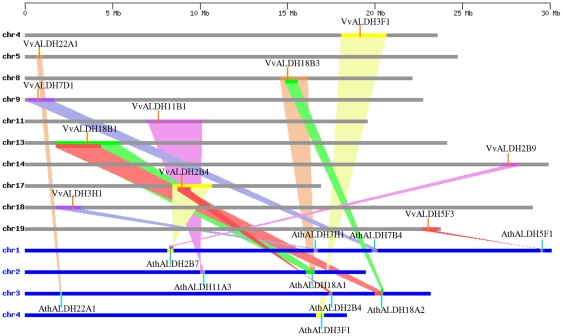
Synteny analysis of *ALDH* genes between grape and *Arabidopsis*. Grape and *Arabidopsis* chromosomes are depicted as horizontal gray and blue bars, respectively. Grape and *Arabidopsis ALDH* genes are indicated by vertical orange and blue lines, respectively. Colored bars denote syntenic regions between grape and *Arabidopsis* chromosomes; the twisted colored bar indicates that the syntenic regions are in reverse orientation.

Large-scale syntenies containing orthologs from seven *ALDH* families (*ALDH2*, *ALDH3*, *ALDH5*, *ALDH7*, *ALDH11*, *ALDH18* and *ALDH22*) in both grape and *Arabidopsis* genomes were identified (Fig. 4). Regarding the single grape-to-*Arabidopsis ALDH* gene correspondences, the syntenies were unambiguous and included the following ortholog pairs: *VvALDH3H1-AthALDH3H1*, *VvALDH7D1-AthALDH7B4*, *VvALDH3F1-AthALDH3F1*, *VvALDH11B1-AthALDH11A3*, *VvALDH5F3-AthALDH5F1* and *VvALDH22A1-AthALDH22A1* (Fig. 4), indicating these genes/families should have been in the genome of last common ancestor of grape and *Arabidopsis*. More challenging for syntenic interpretation are cases where duplicated grape genes corresponded to two *Arabidopsis* genes. These included *VvALDH2B4/VvALDH2B9-AthALDH2B4/AthALDH2B7* and *VvALDH18B3/VvALDH18B1-AthALDH18A1/AthALDH18A2*. *VvALDH6B3* is also located in the syntenic regions, whereas its syntenic *Arabidopsis* counterpart has been lost. The remaining two families (10 and 12) could not be mapped into any synteny blocks. However, we could not conclude that these two families from grape and *Arabidopsis* did not share a common ancestor. This may be explained by the fact that after speciation of grape and *Arabidopsis*, their genomes have undergone multiple rounds of significant chromosomal rearrangement and fusions, followed by selective gene loss, which can severely obscure the identification of chromosomal syntenies.

### Expression profiles of *ALDH* genes under various stress conditions and during berry development and ripening

**Figure 5:**
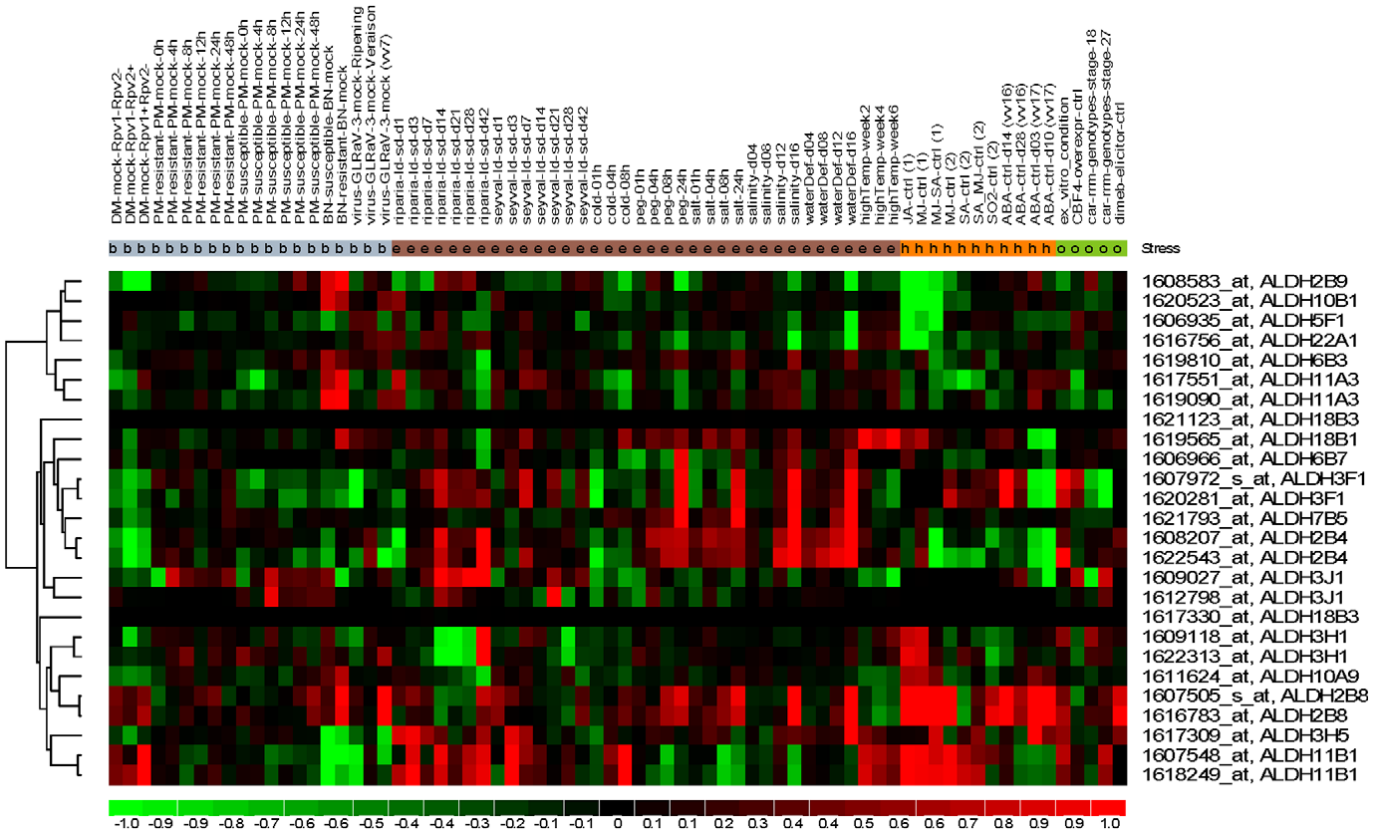
Hierarchical clustering of *ALDH* genes. Details of the experimental conditions are provided in Table S1. Log2 based fold changes was used to create the heatmap. Differences in gene expression changes are shown in color as per the lower scale.

Different approaches have been undertaken to increase plant stress tolerance including manipulating and reprogramming the expression of endogenous stress-related genes. Therefore, identification and functional characterization of potential stressed-related genes provide fundamental information for future improvement of plant stress tolerance. The expression of most plant *ALDH* genes seems to have a common ‘stress response’ pattern within several divergent plant species from mosses to angiosperms [6]. In the present study, we investigated the responses of grape *ALDH* genes to various abiotic and biotic stress conditions as well as their expression patterns during grape berry development and ripening, through mining publicly available grape microarray datasets. A total of 19 experiments containing 430 hybridizations from the Affymetrix grape genome array were obtained. After manual curation, 76 comparisons between different experimental conditions and during berry development were constructed (Table S1). From the Affymetrix grape genome array, we identified 18 *ALDH* genes corresponding to 26 probe sets. Detailed expression of these *ALDH* genes is provided in Table S1. Heatmap representation of expression profiles of these *ALDH* genes is shown in Fig. 5, revealing that a large number of grape *ALDH* genes are highly responsive to certain types of abiotic or biotic stresses.

#### Abiotic stress

Abiotic stresses, such as drought, salinity and extreme temperatures are serious threats to plant growth and crop production. *ALDH* genes play critical roles in the adaptation of plants to various abiotic stresses [4], [35]. The microarray data analyzed included hybridizations generated from plants exposed to polyethylene glycol (PEG), and under cold, high temperature, high salinity and water-deficit stresses.

Drought and salinity are two major environmental factors determining plant productivity and distribution. It has been demonstrated that exposure to drought or salinity leads to the rapid and excessive accumulation of reactive oxygen species (ROS) in plant cells which in turn affects cellular structure and metabolism and homeostasis [36], [37]. ROS induce lipid peroxidation within lipid membranes which generates chemically reactive cleavage products, largely represented by aldehydes [38], [39]. Enhancement of ALDH activity is considered as an efficient strategy to eliminate the toxic aldehydes caused by ROS and oxidative stress [40], [41]. *ALDH* genes that are induced under high salinity and drought conditions have been identified in many plant species, indicating that they may play critical roles in plant adaptation to these stresses [10]. In the present study, our analysis of publicly available microarray datasets indicated that expressions of 13 grape *ALDH* genes were differentially expressed in at least one of the four osmotic treatments (short-term PEG, short-term salinity, long-term salinity and long-term water-deficit) (Table S1). Among them, nine (*VvALDH2B4*, *VvALDH2B8*, *VvALDH3F1*, *VvALDH3H5*, *VvALDH6B3*, *VvALDH6B7*, *VvALDH7B5*, *VvALDH11A3* and *VvALDH18B1*) were up-regulated by long-term salinity and water-deficit treatments; whereas four genes (*VvALDH2B9*, *VvALDH5F1*, *VvALDH11B1* and *VvALDH22A1*) were down-regulated (Table S1). It has been reported that plant *ALDH3* genes may be an important component of ABA-dependent stress response pathways [42]. In addition, both *ALDH3* and *ALDH7* genes are involved in stress-regulated detoxification pathways, and *ALDH7* genes are also turgor-responsive [6], [43]. In *Arabidopsis*, the *ALDH11A3* gene encodes a non-phosphorylating glyceraldehyde-3-phosphate dehydrogenase (GAPDH) which generates NADPH required for biosynthetic processes [6]. However, the exact function of *ALDH11A3* during dehydration and salt stress remains unclear. *ALDH18* genes encode P5CS (Δ1-pyrroline-5-carboxylate synthetase), a key regulatory enzyme that plays a crucial role in proline biosynthesis. Recent studies indicated that *ALDH18* genes were also abiotic stress-responsive [30], [44]. Our findings are largely consistent with studies in *Arabidopsis* and rice that indicated *ALDH* genes from families 2, 3, 7, and 18 showed significant inductions in osmotically stressed plants [10], [11], [30], [35]. However, our analysis identified osmotic stress-induced genes from one additional *ALDH* family (*ALDH*6) in grape. *ALDH6* genes encode methylmalonate semialdehyde dehydrogenases (MM-ALDH, EC 1.2.1.27). Mammalian ALDH6 isozymes play a role in the catabolism of valine and pyrimidines [25]. This enzyme has not been extensively studied in plants but studies have revealed that *ALDH6* is an auxin-responsive gene in rice, implying its possible role in cell differentiation and organ development [45]. Further functional studies are required to reveal the exact role of these genes in grape adaptation to osmotic stress.

Cold stress, which includes chilling (<20°C) and freezing (<0°C) temperatures, adversely affects plant growth and development [46]. Under cold conditions (5°C), two *ALDH* genes (*VvALDH11B1* and *VvALDH18B1*) showed increased expression while five (*VvALDH2B4*, *VvALDH3F1*, *VvALDH10A9*, *VvALDH11A3* and *VvALDH22A1*) showed decreased expression. These seven cold-stress-responsive genes, except *VvALDH10A9*, were also regulated by drought stress, thus there may be a crosstalk between the osmotic- and cold-stress signaling pathways that regulate the expression of grape *ALDH* genes (Table S1). The relationship between *ALDH* gene expression and cold stress has not been previously documented in plants. Our analysis of *ALDH* genes in grape provides initial insights pertaining to cold stress and important candidates for future functional analysis. Under the heat stress, we found that none of the *ALDH* genes displayed significantly changed expression levels (Table S1).

#### Biotic stress

Little attention has been paid to the investigation on expression patterns of *ALDH* genes under biotic stress conditions. It has been shown that *ALDH2* gene expression is regulated by powdery mildew infection in Chinese wild *Vitis pseudoreticulata*, suggesting potential roles of *ALDH* genes during plant pathogen responses [19].

*Plasmopara viticola* is the causal agent of downy mildew, one of the world's most catastrophic and baffling diseases of grapevine. Our microarray data analysis revealed that in a grape line (Rpv1−/Rpv2+) that is highly resistant to *P. viticola*, the expression of eight *ALDH* genes was significantly changed upon the inoculation of *P. viticola*, among which seven (*VvALDH2B4*, *VvALDH2B9*, *VvALDH3F1*, *VvALDH3H1*, *VvALDH6B7*, *VvALDH7B5* and *VvALDH10A9*) were down-regulated and one (*VvALDH11B1*) was up-regulated. In the partially resistant line (Rpv1+/Rpv2−), four *ALDHs* (*VvALDH2B4*, *VvALDH2B9*, *VvALDH7B5*, *VvALDH10A9*) and two (*VvALDH2B8* and *VvALDH11B1*) showed decreased and increased expressions, respectively, upon *P. viticola* infection; while in the susceptible line (Rpv1−/Rpv2−), none of the *ALDH* genes showed significant changes in their expression (Table S1). These results suggested that *ALDHs* could play important roles in the interaction between grapevine and *P. viticola*.

Powdery mildew, caused by an obligate biotrophic fungus, *Uncinula necator* [Schw.] Burr., is another economically important disease of grapevines. Array data analysis results indicated that the expression levels of most *ALDH* genes were not significantly altered upon the *U. necator* infection for both disease-resistant *V. aestivalis* genotype ‘Norton’ and disease-susceptible genotype *V. vinifera* ‘Cabernet sauvignon’. However, one gene, *VvALDH11A3*, was found to be significantly down-regulated at 4 hours post the infection in the disease-susceptible genotype (Table S1), indicating its potential role in powdery mildew development in grapevines.

*Bois Noir phytoplasma* is an emerging disease of *V. vinifera* in several regions of the world. In grape cultivar Manzoni, which is moderately resistant to *Bois Noir phytoplasma*, the expression of two genes (*VvALDH10A9* and *VvALDH11A3*) was significantly increased after infection; while in Chardonnay, a highly susceptible cultivar, three genes (*VvALDH2B4*, *VvALDH3H5* and *VvALDH11B1*) were significantly down-regulated and one gene (*VvALDH6B3*) was up-regulated (Table S1).

Viral diseases also have a serious impact on grapevine productivity and fruit quality. Among the more than 40 different viruses known to infect grapevines, the leaf roll-associated closeter-ovirus-3 (*GLRaV-3*) is one of the most widespread viruses [47]. Berry transcriptomes in two stages of development (veraison and ripening) in cultivar Cabernet Sauvignon infected with *GLRaV-3* were analyzed. The expression of seven *ALDH* genes (*VvALDH2B4*, *VvALDH2B9*, *VvALDH3F1*, *VvALDH3H1*, *VvALDH3H5*, *VvALDH11A3* and *VvALDH11B1*) was significantly decreased in ripening berries when infected with *GLRaV-3*. However, none of the *ALDH* genes showed significantly changed expression in the veraison stage (Table S1).

In summary, our analysis of publicly available array datasets indicated potential roles of *ALDH* genes in plant responses to pathogen infection. Although elucidating exact roles of these *ALDH* genes in plant-pathogen interactions requires further functional analysis, our findings provide a valuable increase in our knowledge base.

#### Hormone treatment

Plant hormones salicylic acid (SA), jasmonic acid (JA) and ethylene (ET) play central roles in biotic stress signaling upon pathogen infection [48]. Methyl Jasmonate (MJ) also affects stress responses and has a well documented role in biotic stress and wounding responses [49]. By contrast, abscisic acid (ABA) is extensively involved in responses to abiotic stresses such as drought, low temperature, and osmotic stress [48]. Analysis of expression data of grape cell-suspension cultures and berries exposed to JA, SA, ABA, MJ, or a combination of SA and MJ indicated that all 18 *ALDH* genes present on the array except *VvALDH6B7* and *VvALDH18B3* showed significantly changed expression in at least one treatment of these signaling molecules (Table S1). The expression of *VvALDH2B8* was induced by all these treatments, suggesting its important role in plant stress tolerances.

ABA plays a key role in plant adaptation to adverse environmental conditions [49]. However, several studies have suggested that both ABA-dependent and ABA-independent regulatory systems are involved in stress-responsive gene expression [50]. The majority of the 13 grape *ALDH* genes showing significantly changed expressions in response to drought or salinity stress were also ABA-responsive. However, two genes (*VvALDH6B3* and *VvALDH7B5*) were apparently not regulated by ABA (Table S1), confirming ABA-independent stress signaling pathways during osmotic responses.

#### Developmental and environmental cues

Grape berry development and ripening is a coordinated regulatory process involving genetically, hormonally, and environmentally controlled interactions of complex gene expression patterns, which ultimately leads to changes in color, texture, flavor, and aroma of the berry. The development and maturation of grape berries has been studied intensely and significant progress has been made during recent years toward elucidating the regulatory networks that determine fruit and wine quality [51]. However, the relationship between grape *ALDH* genes and berry development and maturation has not been reported. Our analysis of microarray data identified a number of grape *ALDH*s whose expression was significantly changed during berry development and ripening, e.g., the expression of *VvALDH2B8*, *VvALDH3H5* and *VvALDH18B1* was significantly increased, while the expression of *VvALDH2B4* and *VvALDH5F1* was significantly decreased during grape berry development and ripening (Table S1), indicating that *ALDH* genes could play important roles in grape berry development.

Day length is an important environmental cue for synchronizing plant growth, flowering, and dormancy with seasonality [52]. We found 15 of 18 grape *ALDH* genes on the array were differentially expressed during long and short photoperiods in either *V. riparia* or *V. spp.* ‘Seyval’, indicating that the expression of *ALDH* genes could be regulated by the photoperiod.

### Conclusion

The aldehyde dehydrogenases (*ALDHs*) comprise a gene superfamily encoding NAD(P)^+^-dependent enzymes that catalyze the irreversible oxidation of a wide range of endogenous and exogenous aromatic and aliphatic aldehydes. Significant progress has been made toward the identification and characterization of *ALDH* gene families in model plants, with little attention paid to *ALDH* gene families in woody species. In the present study we identified 23 *ALDHs* in the *V. vinifera* genome, which were further grouped into ten families, and provided a unified nomenclature for the deduced ALDH polypeptides using the criteria established by the ALDH Gene Nomenclature Committee (AGNC). Our gene structure analysis showed that *ALDHs* from the same families contained highly similar exon-intron structures. Three alternatively spliced transcripts of *ALDH2B4* were also identified. We further showed that segmental and tandem duplications have contributed substantially to the expansion of grape *ALDH* genes. Comparative synteny analysis between *V. vinifera* and *Arabidopsis* genomes showed that the majority of grape and *Arabidopsis ALDH* genes were located in syntenic regions, indicating that these *ALDH* genes had common ancestors. Finally, we analyzed expression profiles of grape *ALDH* genes in responses to various abiotic and biotic stress conditions and during grape berry development, and identified novel candidate *ALDH* genes that are potentially involved in grape tolerances to environmental and biotic stresses and berry development and ripening.

## Methods

### Identification and annotation of grape *ALDH* genes

Previously identified *Arabidopsis* ALDH sequences [6], Pfam domain PF00171 (ALDH family), PS00070 (ALDH cysteine active site), PS00687 (ALDH glutamic acid active site), KOG2450 (aldehyde dehydrogenase), KOG2451 (aldehyde dehydrogenase), KOG2453 (aldehyde dehydrogenase) and KOG2456 (aldehyde dehydrogenase) were used as queries to search in the GenBank non-redundant protein database and the Grape Genome Database (12X) (http://www.genoscope.cns.fr). Protein motifs were additionally queried against the Pfam, PROSITE, and CDD (Conserved Domain Database) [53] databases. The identified grape ALDH proteins were annotated using the criteria established by the ALDH Gene Nomenclature Committee (AGNC) [12]. Briefly, ALDH proteins with amino acid sequences more than 40% identical to previously identified ALDH sequences comprise a family, those with sequences more than 60% identity comprise a subfamily, and those with sequences less than 40% identity represent a new family.

### Sequence alignments and phylogenetic analyses

Multiple alignments of ALDH protein sequences from grape, *Arabidopsis* [6], rice [22], maize [21], *P. patens*, *C. reinhardtii* and *O. tauri* [26], were performed using the ClustalW program [54]. Phylogenetic trees were constructed with the MEGA 4.0 software [55] using the neighbor-joining (NJ) method and the bootstrap test was replicated 1000 times.

### Exon-intron structure analysis of grape *ALDH* genes

The exon-intron structures of grape *ALDH* genes were determined from the alignments of their coding sequences to the corresponding genomic sequences using the est2genome program [56]. The diagrams of exon-intron structures were obtained using the online program FancyGene [57].

### Tandem duplication and synteny analysis

Tandem duplications of *ALDH* genes in the grape genome were identified by checking their physical locations in individual chromosomes. Tandem duplicated genes were defined as adjacent homologous *ALDH* genes on the grape chromosomes, with no more than one intervening gene. For synteny analysis, synteny blocks within the grape genome and between grape and *Arabidopsis* genomes were downloaded from the Plant Genome Duplication Database [58] and those containing grape *ALDH* genes were identified.

### Expression analysis of grape *ALDH* genes

Affymetrix grape microarray data were downloaded from ArrayExpress [59] and PLEXdb [60] databases. A total of 19 experiments were used for our gene expression analyses (Table S1). For each microarray experiment, GCRMA method [61] was applied to perform background adjustment and normalization. The detection calls (present, marginal, or absent) for each probe set were obtained using the mas5calls function in the Affy package [62]. Genes that have absent or marginal calls across the entire arrays of an experiment were not included in the downstream statistical analysis. P-values between treatment and control conditions or during berry development for each experiment were calculated using the Limma package [63] and raw p-values of multiple tests were corrected using the False Discovery Rate (FDR) [64]. Genes with adjusted p-values (FDR) less than 0.05 were identified as differentially expressed genes. Hierarchical clustering of expression profiles of grape *ALDH* genes was performed using dChip [65].

## Supporting Information

Figure S1 **Alternatively spliced transcripts of** ***VvALDH2B4*** **.** (A) Exon-intron structure of alternatively spliced transcripts of *VvALDH2B4*; (B) Alignment of the 5′-open reading frame (ORF) sequences of the three alternative splice variants of *VvALDH2B4*. Translational initiation sites are marked with blue boxes. The 113 bp retained intron of *VvALDH2B4_v2* causes a frame shift in translation and a different translational initiation site.
(PDF)

Table S1 Details of publicly available grape array datasets and grape *ALDH* expression profiles.
(XLS)
