# Supplementary material for: Genome-Wide Identification and Analysis of Grape Aldehyde Dehydrogenase (ALDH) Gene Superfamily
Source: PLoS One. 2012 Feb 15;7(2):e32153. doi: 10.1371/journal.pone.0032153 (PMC3280228; doi:10.1371/journal.pone.0032153)
Supplement: Figure S1 — Alternatively spliced transcripts of VvALDH2B4 . (A) Exon-intron structure of alternatively spliced transcripts of VvALDH2B4; (B) Alignment of the 5′-open reading frame (ORF) sequences of the three alternative splice variants of VvALDH2B4. Translational initiation sites are marked with blue boxes. The 113 bp retained intron of VvALDH2B4_v2 causes a frame shift in translation and a different translational initiation site. (PDF) [file pone.0032153.s001.pdf]

A

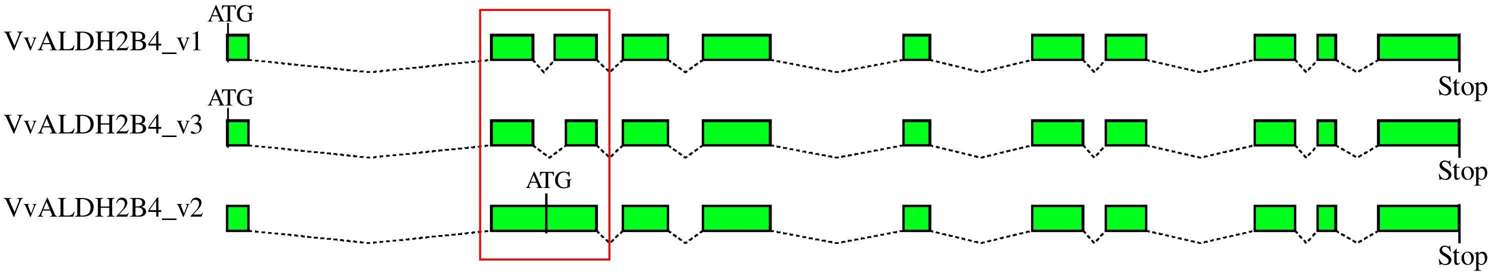

B

```
VvALDH2B4_v1    ATGGCGGCTAGGAGGATCTCCTCGTTGCTCTCTCGTTCTCTCTCTGTCTCTTCTGCTTTTTCTCTTTCTCTAGGCAAAAA    80
VvALDH2B4_v3    ATGGCGGCTAGGAGGATCTCCTCGTTGCTCTCTCGTTCTCTCTCTGTCTCTTCTGCTTTTTCTCTTTCTCTAGGCAAAAA    80
VvALDH2B4_v2    ATGGCGGCTAGGAGGATCTCCTCGTTGCTCTCTCGTTCTCTCTCTGTCTCTTCTGCTTTTTCTCTTTCTCTAGGCAAAAA    80

VvALDH2B4_v1    TTTCAACAGAGGGAAAAGCATCCATCGCTTTAGCACTGCAGCAGCAGCAGCAGTTGAAGAACTCATCACTCCAACTGTTC    160
VvALDH2B4_v3    TTTCAACAGAGGGAAAAGCATCCATCGCTTTAGCACTGCAGCAGCAGCAGCAGTTGAAGAACTCATCACTCCAACTGTTC    160
VvALDH2B4_v2    TTTCAACAGAGGGAAAAGCATCCATCGCTTTAGCACTGCAGCAGCAGCAGCAGTTGAAGAACTCATCACTCCAACTGTTC    160

VvALDH2B4_v1    AGATAAATTACACTCAACTTCTAATTAATGGGCAATTTGTAGATGCAGCATCGG..........................    214
VvALDH2B4_v3    AGATAAATTACACTCAACTTCTAATTAATGGGCAATTTGTAGATGCAGCATCGG..........................    214
VvALDH2B4_v2    AGATAAATTACACTCAACTTCTAATTAATGGGCAATTTGTAGATGCAGCATCGGGTAATCATTTTTGTGTGGCAATGTT    240

VvALDH2B4_v1    ................................................GAAAAACATTCCCCACCTTTGACCCTCGCACG    246
VvALDH2B4_v3    ................................................................................    214
VvALDH2B4_v2    TCCATCATTAATGAATCATGTATGAAACCGATGTCGGTATCATTACAGGAAAAACATTCCCCACCTTTGACCCTCGCACG    320

VvALDH2B4_v1    GGAGAAGTGATTGCTAATGTTGCAGAAGGTGATGCAGAAGATATCAATCGGGCAGTGTCCGCTGCTCGAAAGGCGTTTGA    326
VvALDH2B4_v3    .......TGATTGCTAATGTTGCAGAAGGTGATGCAGAAGATATCAATCGGGCAGTGTCCGCTGCTCGAAAGGCGTTTGA    287
VvALDH2B4_v2    GGAGAAGTGATTGCTAATGTTGCAGAAGGTGATGCAGAAGATATCAATCGGGCAGTGTCCGCTGCTCGAAAGGCGTTTGA    400

VvALDH2B4_v1    TGAAGGACCATGGCCAAGGATGAGCCCCTATGAAAGGTCGCGGATATTGTTGCGGTTTGCTGATTTAGCCGAGAAACACA    406
VvALDH2B4_v3    TGAAGGACCATGGCCAAGGATGAGCCCCTATGAAAGGTCGCGGATATTGTTGCGGTTTGCTGATTTAGCCGAGAAACACA    367
VvALDH2B4_v2    TGAAGGACCATGGCCAAGGATGAGCCCCTATGAAAGGTCGCGGATATTGTTGCGGTTTGCTGATTTAGCCGAGAAACACA    480

VvALDH2B4_v1    ATGATGAGCTTGCAGCTCTA    426
VvALDH2B4_v3    ATGATGAGCTTGCAGCTCTA    387
VvALDH2B4_v2    ATGATGAGCTTGCAGCTCTA    500
```

Figure S1 Alternatively spliced transcripts of *VvALDH2B4*. (A) Exon-intron structure of alternatively spliced transcripts of *VvALDH2B4*; (B) Alignment of the 5'-open reading frame (ORF) sequences of the three alternative splice variants of *VvALDH2B4*. Translational initiation sites are marked with blue boxes. The 113 bp retained intron of *VvALDH2B4_v2* causes a frame shift in translation and a different translational initiation site.
